# Hamman’s syndrome in a patient with uncontrolled type 1 diabetes mellitus: a case report

**DOI:** 10.1186/s40981-025-00792-x

**Published:** 2025-05-17

**Authors:** Asahi Ishihara, Katsuyuki Sagishima, Tadashi Ejima, Manami Kuwahara, Naoyuki Hirata

**Affiliations:** 1 Department of Critical Care Medicine, Kumamoto University Hospital, 1-1-1, Honjo, Chuo-Ku, Kumamoto, 860-8556 Japan; 2 Department of Anesthesiology, Kumamoto University Hospital, 1-1-1, Honjo, Chuo-Ku, Kumamoto, 860-8556 Japan

**Keywords:** Hamman’s syndrome, Diabetic ketoacidosis, Pneumomediastinum

## Abstract

**Background:**

Hamman’s syndrome is a clinical entity characterized by the spontaneous leakage of air into the mediastinum. We report a patient with Hamman’s syndrome associated with diabetes ketoacidosis (DKA).

**Case presentation:**

A 20-year-old foreign technical intern visited to a hospital because of nausea and shortness of breath. He had been diagnosed with diabetes in his home country and had initiated insulin therapy; however, since arriving in Japan, he had not accessed any medical services. Computed tomography revealed pneumomediastinum, while laboratory tests showed marked hyperglycemia, metabolic acidosis, and a significantly elevated blood ketone level (15,044 µmol/L). The patient was diagnosed with Hamman’s syndrome associated with DKA. Upper gastrointestinal endoscopy showed no evidence of gastrointestinal perforation. Conservative intensive care, including insulin therapy and fluid resuscitation, resulted in clinical improvement.

**Conclusion:**

This case highlights the importance of recognizing Hamman’s syndrome in DKA and the need for culturally competent care in international residents.

## Background

Spontaneous pneumomediastinum associated with diabetic ketoacidosis (DKA) was first reported by Hamman in 1937 [1] and is now referred to as Hamman’s syndrome. It is hypothesized that repeated vomiting, persistent deep and rapid respiration (Kussmaul breathing) due to DKA increase intrathoracic pressure, allowing air to escape into the mediastinum through alveolar rupture leading to pneumomediastinum [1–3]. Development of DKA and mediastinitis resulted from pneumomediastinum can lead to critical conditions; therefore, patients with Hamman’s syndrome require intensive care management. This report presents a case of Hamman’s syndrome that improved with intensive care management, including glycemic control, antibiotic therapy, and continuous monitoring.

## Case presentation

The patient was a 20-year-old Muslim male of East Asian descent (160 cm, 43.5 kg) who had arrived in Japan 2 months prior to admission to work as a technical intern trainee in the construction industry. He had been diagnosed with type 1 diabetes mellitus (T1DM) in his home country 2 months earlier and had initiated insulin therapy at that time. However, he had not sought any medical care since his arrival in Japan. He reported self-administering insulin once daily, although the specific type and dosage were unknown. There was no history of oral hypoglycemic agents, drug or food allergies, or any relevant family medical history.

The patient developed nausea followed by multiple episodes of vomiting, accompanied by coughing and shortness of breath, prompting him to seek medical attention at a local hospital. CT revealed the presence of pneumomediastinum, and arterial blood gas analysis demonstrated severe acidemia. Due to the need for intensive care management and the possibility of surgical intervention, he was urgently transferred to the intensive care unit of our hospital.

**Fig. 1:**
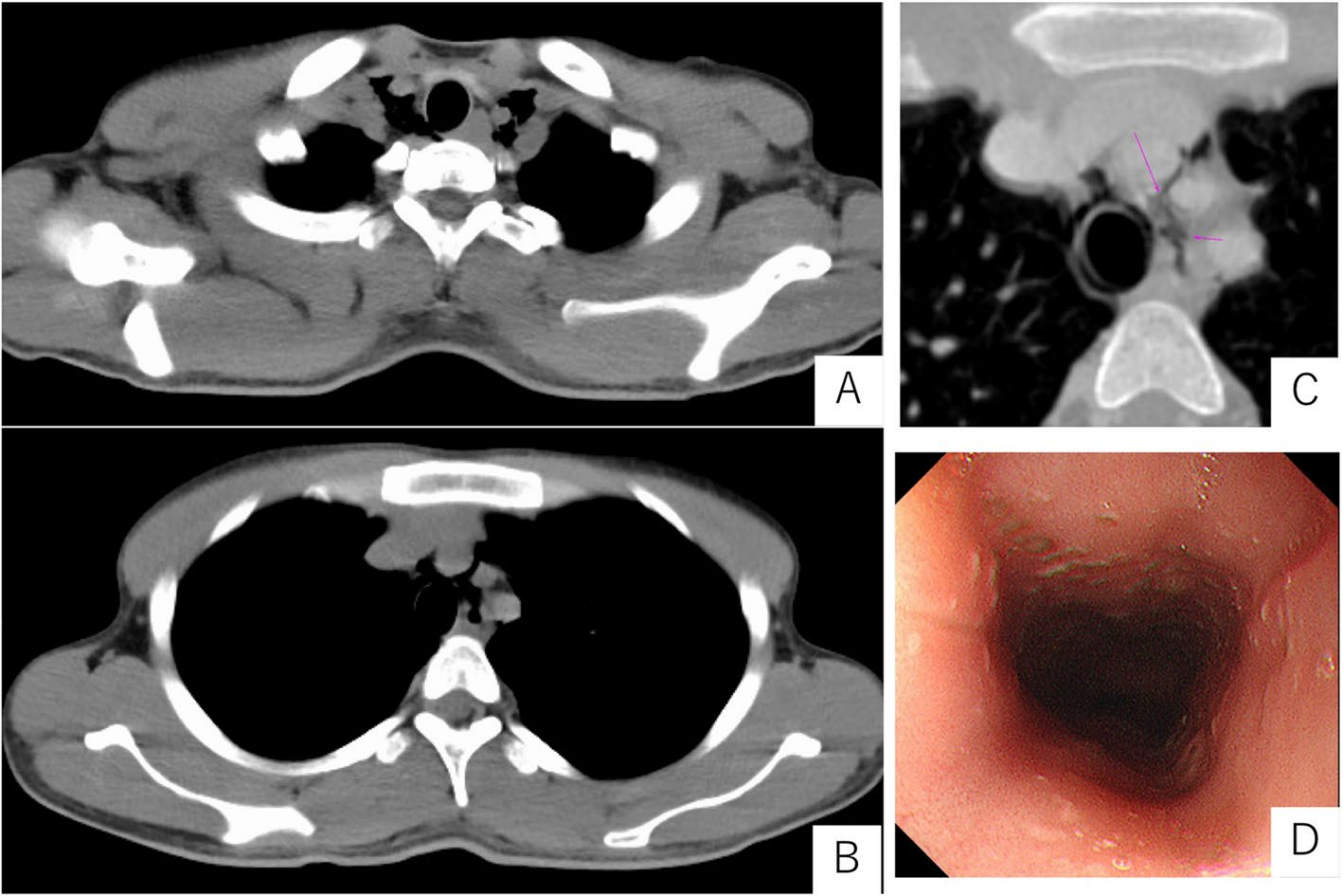
Computed tomography (**A**–**C**) and an image of upper gastrointestinal endoscopy after admission to our hospital

**Fig. 2:**
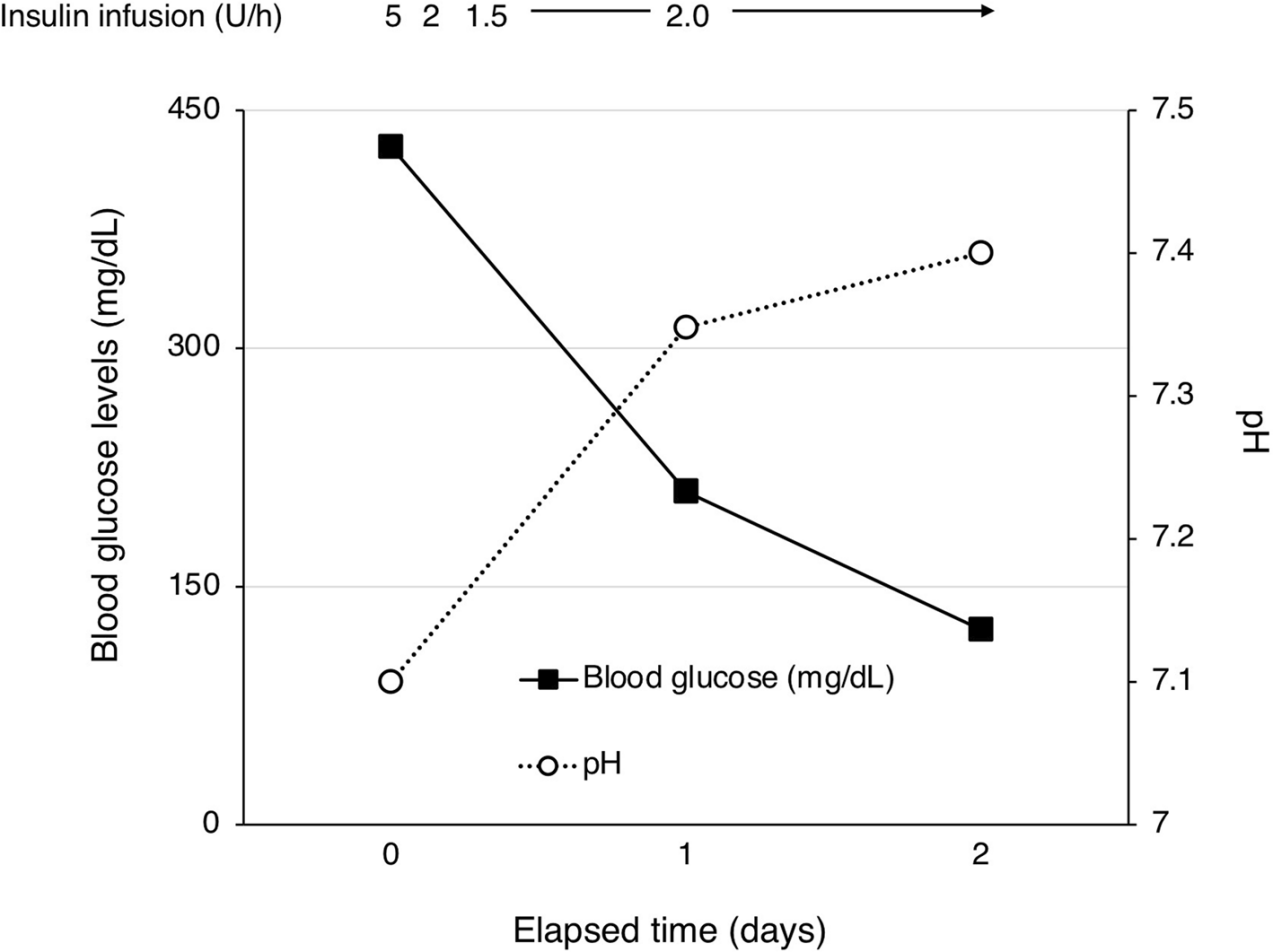
Changes in blood glucose levels and pH of arterial gas analysis

**Fig. 3:**
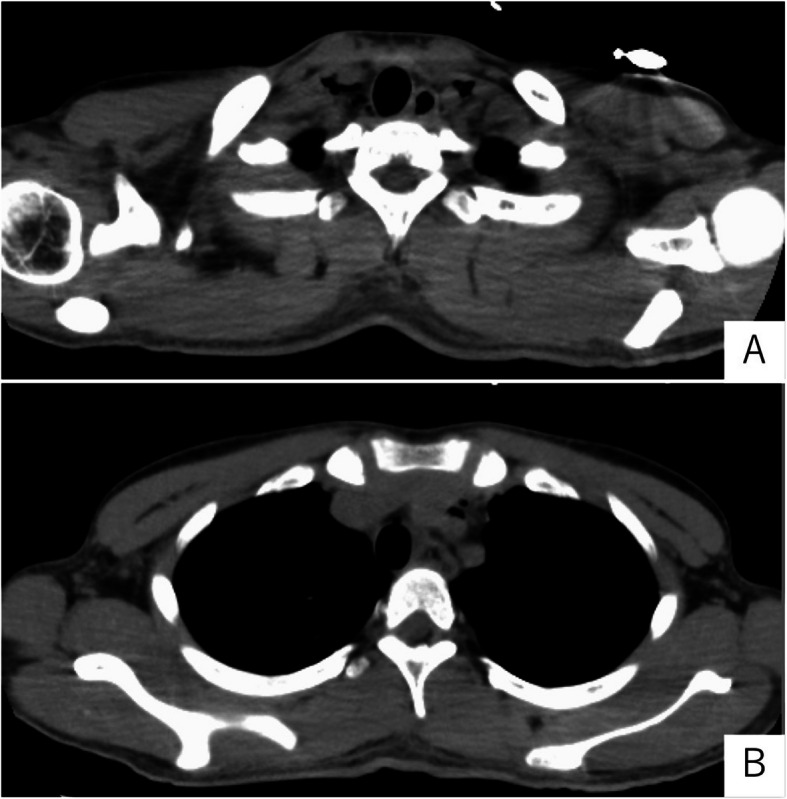
Follow-up computed tomography performed on hospital day 3

Upon admission to our intensive care unit, the patient's neurological status was as follows: Glasgow Coma Scale score E3 V5M6. Pupils were equal and reactive to light, measuring 2.5 mm bilaterally. Vital signs were as follows: body temperature, 37.6 °C; heart rate, 97 beats per minute with a sinus rhythm; blood pressure, 129/74 mmHg; respiratory rate, 23 breaths per minute; and oxygen saturation (SpO₂), 98% on room air. Cardiac examination revealed no murmurs, and bilateral breath sounds were clear. Subcutaneous emphysema was noted extending from the cervical region to the anterior chest. Laboratory findings on admission were indicative of DKA, with an HbA1c of 17.1%, blood glucose level of 427 mg/dL and serum ketone bodies elevated to 15,044 µmol/L. Arterial blood gas analysis demonstrated severe metabolic acidosis, with a pH of 7.100, bicarbonate (HCO₃⁻) level of 3.1 mmol/L, and a markedly negative base excess of − 23.9 mmol/L. Partial pressure of carbon dioxide (pCO₂) was decreased to 10.3 mmHg, indicating respiratory compensation. The arterial oxygen level (pO₂) was 126.7 mmHg on room air, and lactate was within the normal range at 1.35 mmol/L. These findings are consistent with a diagnosis of DKA with severe acid–base disturbance. Although chest radiography did not clearly demonstrate subcutaneous emphysema or pneumomediastinum, CT (Fig. 1A–C) confirmed the presence of pneumomediastinum. A discontinuity in the anterior esophageal wall at the upper thoracic level raised suspicion for esophageal perforation. While upper gastrointestinal endoscopy did not reveal a definitive site of perforation (Fig. 1D), oral intake was withheld to prevent the development of mediastinitis, and piperacillin/tazobactam (PIPC/TAZ) was initiated. For the management of DKA, fluid management and continuous intravenous insulin infusion were continued, resulting in improvements in both hyperglycemia and acidemia (Fig. 2). Follow-up CT performed on hospital day 3 (Fig. 3A, B) showed a reduction in pneumomediastinum. As there were no signs of overt infection, PIPC/TAZ was discontinued. The patient’s insulin therapy was transitioned to subcutaneous insulin glargine (0–0–15 units), and he was transferred to the general ward. On hospital day 7, an upper gastrointestinal contrast study showed no abnormalities, and oral intake was resumed that evening. Due to religious and dietary preferences, his food intake remained inconsistent, making insulin dose adjustment challenging. Eventually, glycemic control was achieved with insulin lispro (8–6-6 units) and insulin glargine (0–0–8 units), resulting in fasting glucose levels around 200 mg/dL and postprandial levels between 200 and 350 mg/dL. Although glycemic control was not fully optimized, the patient’s overall condition was stable, and continued treatment on an outpatient basis was considered feasible.

Given his plan to return to construction work as a technical intern trainee, insulin doses were adjusted according to anticipated physical activity: insulin lispro (4–3-3 units) and insulin glargine (0–0–6 units). He was discharged home on hospital day 12, with outpatient follow-up arranged at a nearby clinic.

## Discussion

We reported a case of Hamman’s syndrome, which is defined as pneumomediastinum associated with DKA, and was managed successfully with intensive care management, including glycemic control, antibiotic therapy, and continuous monitoring.

This case is unique in that the patient was a foreign technical intern with limited access to healthcare and religious dietary restrictions, which affected both the development and management of DKA. These sociocultural factors are rarely discussed in previous reports of Hamman’s syndrome, making this case particularly relevant in today’s increasingly multicultural medical environment.

In addition to Hamman’s syndrome, Boerhaave syndrome is also a known condition in which vomiting or respiratory effort can lead to pneumomediastinum [4]. Boerhaave syndrome is characterized by full-thickness esophageal tears or perforation, often accompanied by pleural effusion, shock, and signs of sepsis. In the present case, no definitive esophageal perforation was observed. Therefore, a diagnosis of Hamman’s syndrome was made, and conservative treatment was selected.

Repeated vomiting and persistent deep, rapid respiration (Kussmaul breathing) associated with DKA can contribute to the development of spontaneous pneumomediastinum [2, 3]. Kussmaul breathing significantly increases intrathoracic pressure swings. These repetitive pressure changes, combined with vomiting-induced strain, can result in alveolar rupture, allowing air to escape along bronchovascular sheaths into the mediastinum. This mechanism underscores the cardiorespiratory stress involved in DKA, particularly when compounded by delayed treatment and poor insulin adherence [2, 3].

This case is further notable in that, despite initial suspicion of esophageal perforation, conservative management including fasting, broad-spectrum antibiotics, and close monitoring was successfully employed. Although surgical intervention has been reported in some cases [5], most resolve with non-invasive treatment [3, 6–8]. Multidisciplinary collaboration between intensive care, infectious disease, gastroenterology, and surgical teams was essential to safely navigate the decision for non-operative management.

## Conclusion

This case illustrates Hamman’s syndrome in a young patient with untreated T1DM and DKA, complicated by sociocultural factors. Conservative treatment, intensive monitoring, and multidisciplinary care resulted in a favorable outcome. The case highlights the importance of developing individualized care strategies that take into account patients’ social and cultural backgrounds, particularly in the management of chronic diseases and their complications among international or underserved populations.

## Data Availability

Data sharing not applicable to this article as no datasets were generated or analyzed during the current report.

## Abbreviations

DKA: Diabetic ketoacidosis
CT: Computed tomography
T1DM: Type 1 diabetes mellitus
